# Exogenous carbohydrate and regulation of muscle carbohydrate utilisation during exercise

**DOI:** 10.1007/s00421-021-04609-4

**Published:** 2021-02-05

**Authors:** James J. Malone, Andrew T. Hulton, Don P. M. MacLaren

**Affiliations:** 1 School of Health Sciences, Liverpool Hope University, Taggart Avenue, Liverpool, L16 9JD UK; 2 Department of Nutritional Sciences, University of Surrey, Guildford, UK; 3 Research Institute for Sport and Exercise Sciences, Liverpool John Moores University, Liverpool, UK

**Keywords:** Infusion, Metabolism, Barriers, Sport, Nutrition, Performance

## Abstract

**Purpose:**

Carbohydrates (CHO) are one of the fundamental energy sources during prolonged steady state and intermittent exercise. The consumption of exogenous CHO during exercise is common place, with the aim to enhance sporting performance. Despite the popularity around exogenous CHO use, the process by which CHO is regulated from intake to its use in the working muscle is still not fully appreciated. Recent studies utilizing the hyperglycaemic glucose clamp technique have shed light on some of the potential barriers to CHO utilisation during exercise. The present review addresses the role of exogenous CHO utilisation during exercise, with a focus on potential mechanisms involved, from glucose uptake to glucose delivery and oxidation at the different stages of regulation.

**Methods:**

Narrative review.

**Results:**

A number of potential barriers were identified, including gastric emptying, intestinal absorption, blood flow (splanchnic and muscle), muscle uptake and oxidation. The relocation of glucose transporters plays a key role in the regulation of CHO, particularly in epithelial cells and subsequent transport into the blood. Limitations are also apparent when CHO is infused, particularly with regards to blood flow and uptake within the muscle.

**Conclusion:**

We highlight a number of potential barriers involved with the regulation of both ingested and infused CHO during exercise. Future work on the influence of longitudinal training within the regulation processes (such as the gut) is warranted to further understand the optimal type, dose and method of CHO delivery to enhance sporting performance.

## Introduction

Carbohydrates (CHO) and fats are the two major energy sources that fuel muscle during prolonged steady state and intermittent exercise. The fatigue associated with prolonged performance has been reported in some early classical studies to coincide with the depletion of endogenous stores of CHO (Bergström and Hultman 1967), and/or of hypoglycaemia (Christensen and Hansen 1939). More recently, Cermack and Loon (2013) have explored and collated studies which demonstrate similar overall conclusions. Significant improvements in endurance performance and capacity are well established when CHO is ingested before and/or during activity (Stellingwerff and Cox 2014). These improvements could be due to a number of factors such as stimulation of CHO receptors in the oral cavity and thereby modulating neural drive and attenuating perceived exertion (Carter et al. 2004), and/or maintenance of plasma glucose concentration leading to an increase in CHO oxidation late in exercise (Coggan and Coyle 1989; Jeukendrup 2004). In addition, CHO intake during exercise not only increases oxidation but may spare the use of the limited muscle glycogen and thereby improve performance or capacity (Stellingwerff et al. 2007; Tsintzas et al. 1995), although not universally accepted as a number of studies have failed to show a sparing effect on muscle glycogen (Coyle et al. 1986; Mitchell et al. 1989).

Stellingwerff and Cox (2014) proposed a likelihood of performance benefits with CHO ingestion when exercise was longer than 2-h but not necessarily if the bout was less than 1-h. They concluded that the primary mechanism by which CHO enhances endurance performance was due to a high rate of CHO delivery resulting in elevated rates of CHO oxidation. Consequently, numerous investigations have explored the promotion of CHO delivery to the muscle using high levels of a single source of CHO or by ingesting multiple transportable CHO such as glucose: fructose combinations (Newell et al. 2018). The issue with ingesting large amounts of CHO during performance (particularly running and cycling to a lesser extent) is that the gastrointestinal system is compromised and may lead to unwarranted symptoms such as gut pain, flatulence, diarrhea, and vomiting. Even so, it appears that the maximum rate of exogenous CHO is achieved when ingesting around 90 g/h. Amounts of ingested CHO at these high levels results in a maximal rate of exogenous CHO oxidation of ~ 1.0 g/min for single sources of CHO or ~ 1.75 g/min using multiple transportable CHO (Jeukendrup 2010).

Studies whereby glucose delivery to the muscle during exercise is via infusion invariably results in higher levels of total CHO oxidation (i.e. ~ 1.4–2.5 g/min) than can be achieved by ingestion (MacLaren et al. 1999; Mohebbi et al. 2020). It is quite likely therefore that there are significant impediments regarding CHO oxidation vis a vis ingestion of glucose/CHO in comparison with an infusion of glucose. This review briefly explores the likely causes of impaired glucose delivery to muscle during exercise from ingested and infused sources, the rates of both total CHO oxidation as well as exogenous glucose oxidation during exercise, before examining some regulatory considerations within muscle.

## Likely ‘barriers’ of exogenous CHO ingestion: utilisation and oxidation

**Fig. 1:**
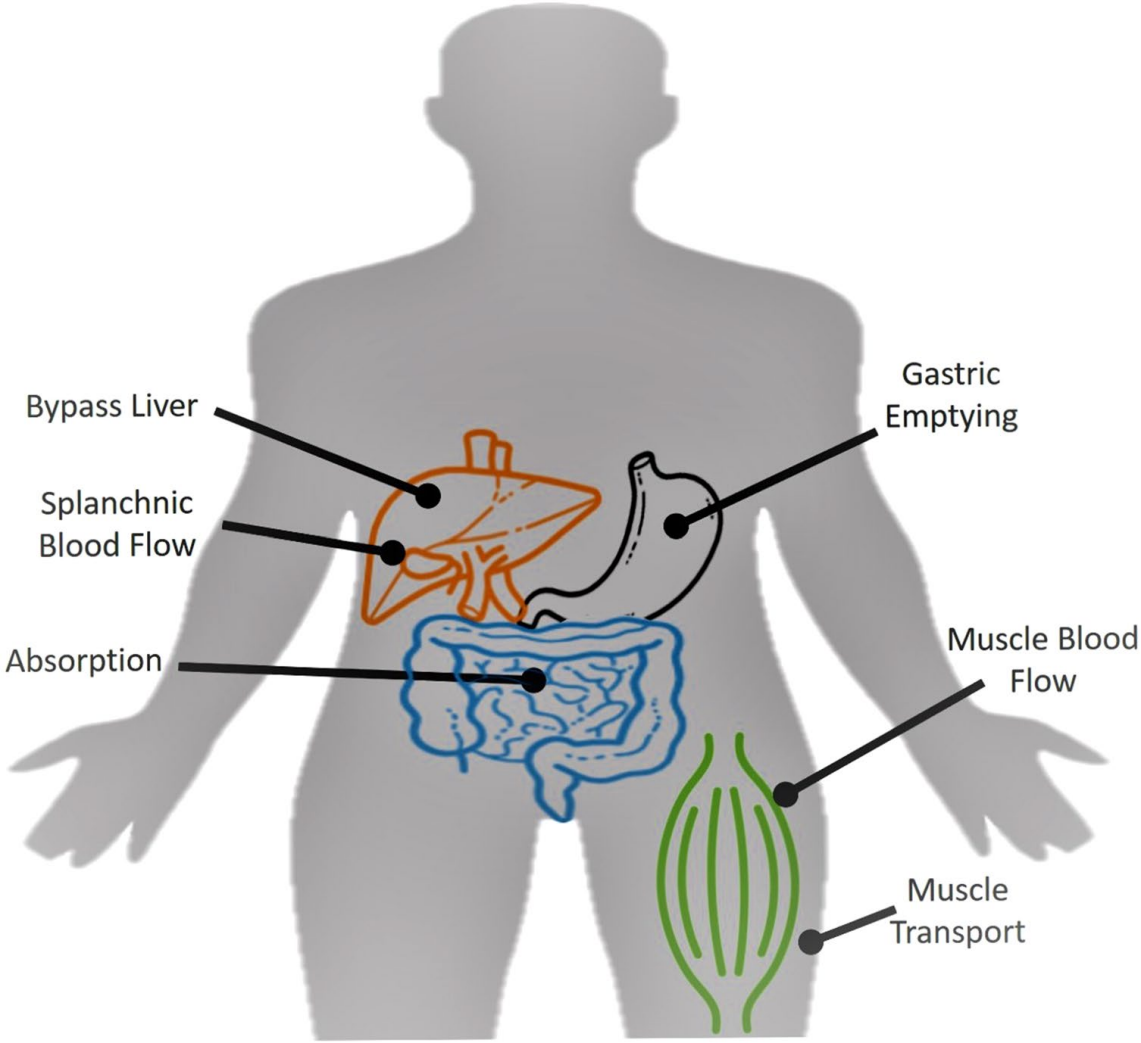
Potential ‘barriers’ to exogenous CHO oxidation

When CHO are ingested during exercise, the source passes through the mouth, stomach and into the small intestine where digestion is complete and absorption takes place. The monosaccharide that then enters into the hepatic portal system passes through the splanchnic bed and then into general circulation where it arrives at the muscles cells for oxidation. It is highly unlikely that CHO is absorbed across the mouth and so is unlikely to affect oxidation. Indeed, the so-called mouth rinsing studies have not shown any changes in CHO oxidation during exercise bouts lasting between 60 and 90 min (Wright and Davison 2013). Consequently, the potential ‘barriers’ to CHO oxidation from ingested sources are the stomach (gastric emptying), the small intestine (gut absorption), passage through the hepatic portal system, splanchnic blood flow, delivery to the muscle (blood flow), transport across the muscle membrane, and entry into and oxidation by the mitochondria (see Fig. 1).

Previous research has demonstrated that the energy content and osmolality of the ingested solution plays a key role in the rate of gastric emptying (Vist and Maughan 1994). Solutions of low osmolality empty from the stomach at a faster rate than those with a high osmolality. Beverages with as little as 2.5% CHO have been shown to empty more slowly than water (Shi et al. 1995). The amount of CHO delivery to the intestine and the rate of exogenous CHO oxidation increases linearly with increasing CHO concentration despite the decrease in gastric emptying, although only solutions with low or isotonic CHO content should be imbibed during prolonged exercise as they are emptied more rapidly and help hydrate. When the requirement is for a greater amount of CHO during strenuous exercise, this can be achieved with a more concentrated CHO source irrespective of the reduced gastric emptying (Foster 1990). The type of CHO ingested appears immaterial for gastric emptying since osmolality is more important (El-Sayed et al. 1997).

**Fig. 2:**
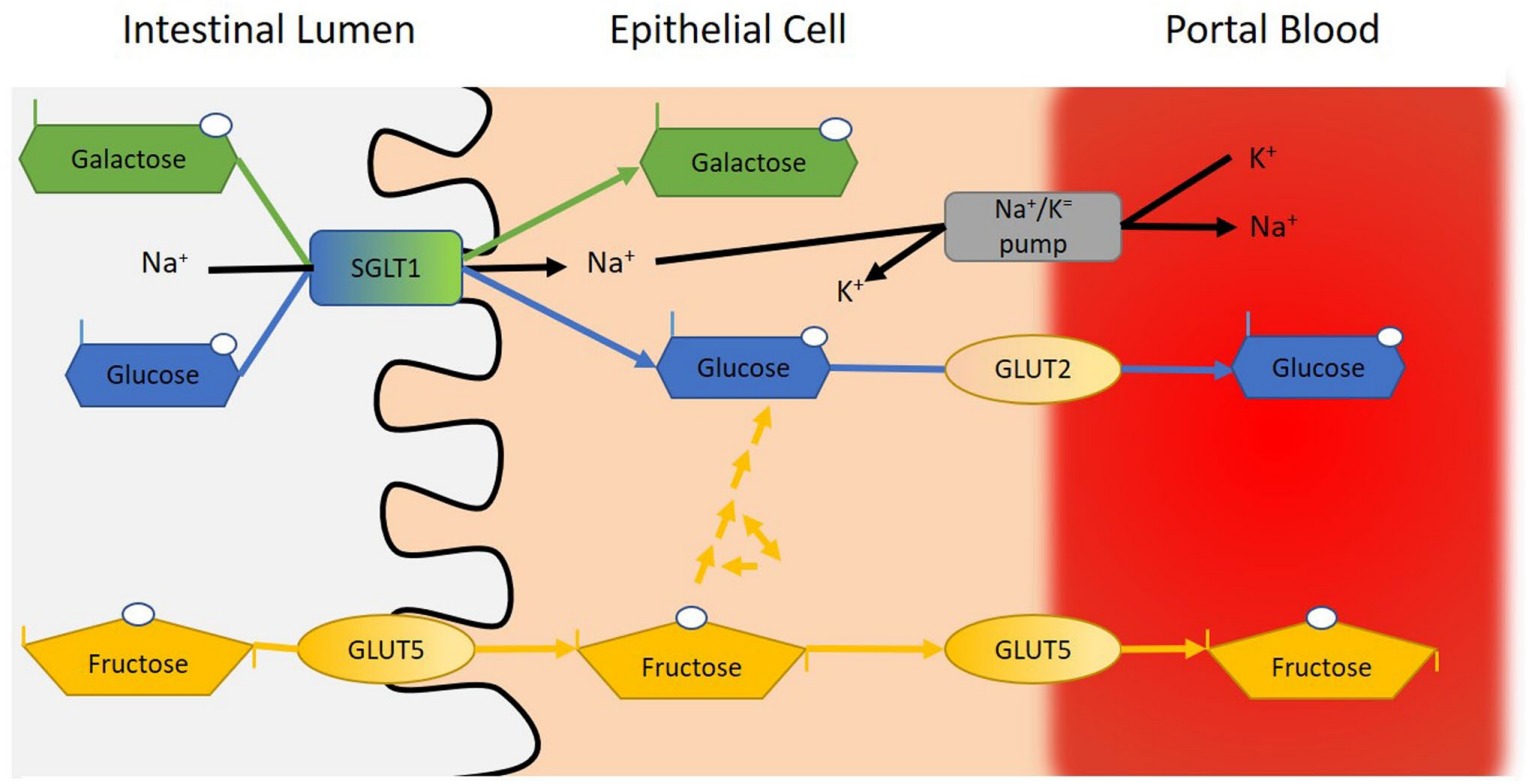
Schematic illustrating the uptake of glucose, galactose, and fructose across the intestinal cells and into splanchnic circulation

The majority of CHO drinks ingested during exercise are monosaccharides or so-called simple sugars such as glucose, fructose, and galactose, although disaccharides such as sucrose, and polysaccharides such as maltodextrins and even starch have been employed. The disaccharides and polysaccharides are required to be digested to their respective monosaccharides before absorption across the gut in the small intestine can occur. It is established that a sodium-dependent glucose transporter (SGLT1) and a glucose transporter (GLUT 5) are required for glucose/galactose and fructose uptake, respectively, across the brush border, and that GLUT2 is required to transport the monosaccharides into the portal blood vessels (Goodman 2010). Evidence is available that the number of SGLT1 transporters are in abundance when compared with GLUT5 transporters, and is a factor as to why glucose uptake across the intestine is greater (and faster) than fructose (Jeukendrup 2017). Indeed, high concentrations of fructose ingested during running-based activities, in particular, have been reported to contribute to increases in gastrointestinal problems – notably diarrhea, abdominal pain, and flatulence (Prado de Oliveira et al. 2014). Figure 2 illustrates the uptake of glucose, galactose, and fructose across the intestinal cells and into splanchnic circulation. Although beyond the scope of this review, high CHO dietary intake has been shown to upregulate SGLT1 transporters in mice (Ferraris et al. 1992). To date, no studies have explored this within a human cohort.

The interplay between exercise, the gut and CHO oxidation was the focus of an interesting paper by Rehrer et al. (1992), in which participants cycled at 70% ${\dot{\text{V}}}$O_2max_ for 80-min. Drinks were consumed at 0, 20, 40, and 60 min and included water, 4.5% glucose, 17% glucose, and 17% maltodextrin. The CHO drinks were enriched with ^13^C to measure exogenous CHO oxidation. Gastric volume was measured at 80 min. The total amount of CHO ingested over the 80-min were 58.2 g, 1220 g, and 1220 g for the 4.5% glucose solution, 17% glucose solution, and 17% maltodextrin solution, respectively, whilst the amount emptied from the stomach (gastric emptying) was 55 g, 132.7 g, and 146.9 g, respectively. The oxidation of the exogenous CHO accounted for 31.5 g, 42 g, and 39.1 g. Much of the ingested CHO was not oxidized. The discrepancy between amounts ingested and amounts oxidized could not account for a delay in gastric emptying. There were large differences in the total amount of CHO being emptied from the stomach with the different treatments, but the differences in total exogenous CHO utilized were small in comparison. When the oxidation data are described in terms of percent CHO ingested that was emptied, the discrepancy between the amount of CHO emptied and the amount oxidized was greatest in the 17% solutions. With the 4.5% solution, 57% of the CHO that was emptied was oxidized, in comparison to the l7% glucose and 17% maltodextrin solutions with only 32% and 27%, respectively, of the amount emptied was oxidized.

In a seminal paper, Ahlborg and Felig (1976) calculated that approximately 50% of a 200 g bolus of CHO consumed during 3-h of exercise ‘escaped the splanchnic bed’. They concluded that even in the face of the stimulatory effects of exercise on hepatic glycogenolysis and gluconeogenesis, the liver remains the major site of retention and disposal of an ingested glucose meal. This means that under such circumstances only 50% of the eaten CHO would be available for oxidation by muscle. However, the exercise intensity was only at 40% ${\dot{\text{V}}}$O_2peak_ and so the findings should be treated with caution. More recent observations are that around 66% of glucose absorbed during exercise escapes the first-pass liver uptake (~ 33% is retained) and is available for muscle delivery and oxidation, whereas nearly 100% of fructose is retained by the liver (Tappy and Le 2010). Fructose is taken up by the liver and undergoes either oxidation or is converted to glucose and lactate, which are then transported from the liver for muscle and other tissue to utilize.

During exercise there is a reduction in splanchnic blood flow (Knight et al. 2017) and so, in general, the availability of absorbed nutrient sources may be further compromised. This is another possible factor to consider vis a vis utilisation of ingested CHO. Once ingested glucose is in the general circulation, it can be taken up into muscle for oxidation. Glucose does not freely diffuse into muscle, rather it is taken across the plasma membrane using a glucose transporter (GLUT4), which normally resides in intracellular vesicles and is translocated to the plasma membrane as a consequence of signaling mechanisms. A brief description of glucose transport across the sarcolemma is discussed later in the present review. Consequently, on reflection, the CHO ingested has to empty from the stomach rapidly, be digested and reduced to monosaccharides if in a complex form, get absorbed across the gut wall, pass into the body circulatory system from the splanchnic region, and then get transported across the muscle membrane before oxidation is possible. Highlighting a magnitude of barriers and potential limiting factors preceding the utilisation of CHO during exercise (Rosset et al. 2017).

The maximal rates of exogenous glucose oxidation during exercise have consistently observed to be approximately 1.0 g/min irrespective of the dose ingested above 100 g (Jeukendrup 2010). Ingestion of disaccharides and short-chain glucose polysaccharides such as maltose and maltodextrins result in similar maximal oxidation rates as glucose. Since glucose polysaccharides are required to be digested before absorption and yet maximal rates of oxidation are similar to glucose, this would indicate that pre-absorptive factors are not limiting. Consequently, the limitation of CHO oxidation maybe considered to be at the level of intestinal absorption, with the ≈1 g/min plateau being consistent with intestinal glucose absorption kinetics. This hypothesis was primarily based on multiple intestinal segmentations experiments showing limited absorption of concentrated glucose solutions (Shi et al. 1995). Another physiological effect of exercise, decreased splanchnic blood flow, may also limit intestinal absorption capacity. Yet, in absence of invasive direct assessments of glucose flows across the intestinal barrier, the idea that intestinal absorption limits exogenous glucose oxidation during exercise remains a hypothesis.

The plateau in exogenous glucose oxidation may also result from hepatic limitations. The route for ingested CHO is to follow portal circulation to the liver, where they can either be stored, metabolized or pass to the systemic circulation. The liver is also known to play a pivotal role in the maintenance of euglycemia through releasing the precise amount of glucose required to match extrahepatic use (Moore et al. 2012). Hence, the factors responsible for the limitation in exogenous glucose oxidation during exercise remain unclear, but probably not restricted to intestinal glucose absorption. For a more comprehensive treatise on the matter of the CHO intake, the gastrointestinal tract and exercise, it is worth reading Rosset et al. (2017).

Since the gut presents a ‘barrier’ not just in terms of CHO delivery into the blood but also in relation to gastrointestinal problems, any question as to the maximal potential rates of exogenous CHO utilization during exercise are thereby hindered by the gut. However, infusing glucose directly into a vein disposes of the need for gut transport and other inherent problems. Previous work in which we have been involved using the hyperglycemic glucose clamp technique to observe metabolic changes during intense bouts of exercise, clearly demonstrated that maintained hyperglycemia (by glucose infusion) resulted in a maximal glucose utilisation rate (GUR) of 1.8 g/min (i.e. 108 g/h) and a maximal rate of total CHO oxidation of 2.7 g/min (MacLaren et al. 1999). Therefore, ~ 70% of the exogenous CHO was oxidized; the rest of the CHO oxidation arising from endogenous sources (most probably muscle glycogen). In fact, two of our younger participants presented with a GUR of ~ 2.8 g/min (168 g/h) which is similar to data we reported more recently (Mohebbi et al. 2020). It would thus be reasonable to suggest that the ~ 1 g/min higher rate of exogenous glucose use from infusion compared with ingestion studies is, in part, due to the gut presenting as a ‘barrier’.

## Glucose delivery to muscle

**Fig. 3:**
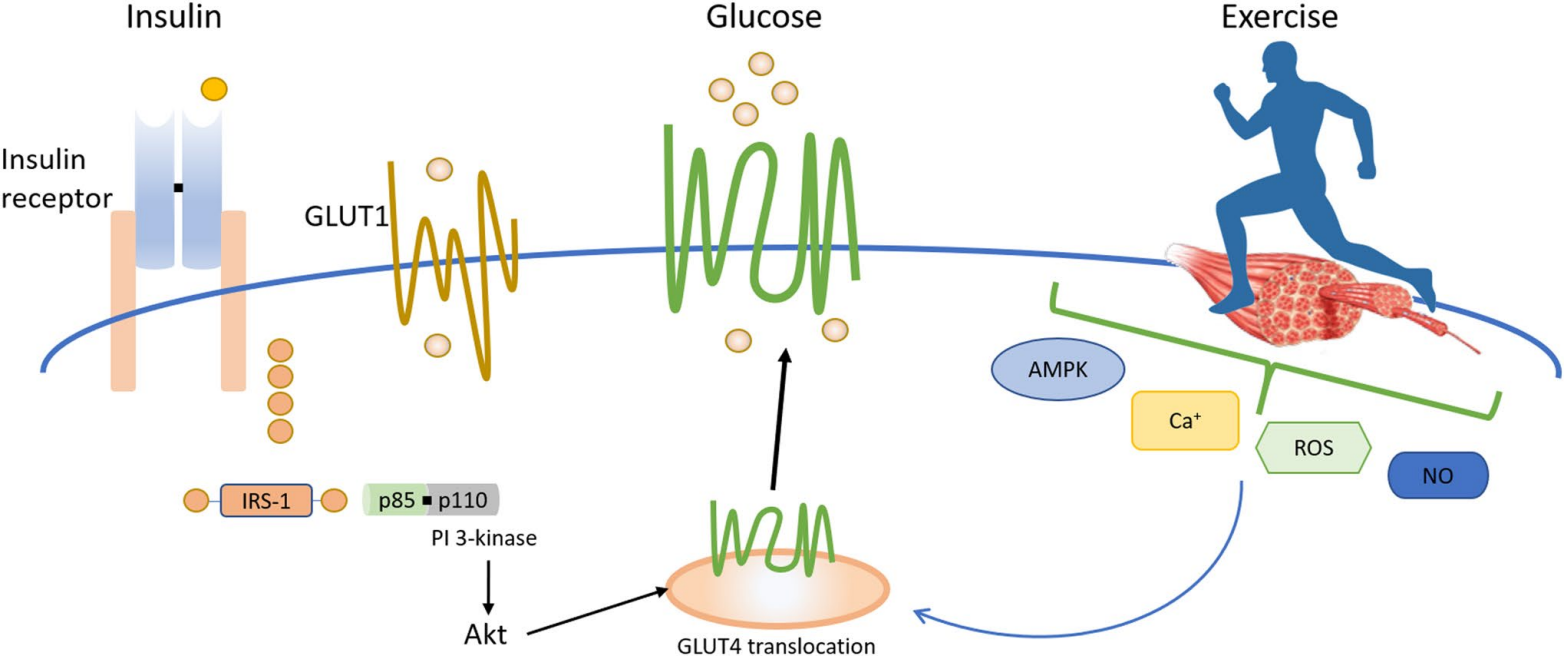
Insulin and exercise stimulated GLUT4 translocation to the cell membrane in skeletal muscle

For ingested CHO sources we can appreciate there are significant ‘barriers’ in terms of uptake across the gut and movement into circulation. However, there remain further ‘barriers’ to transport the glucose into the muscle for disposal. These ‘barriers’ are most notable for studies in which glucose is infused, but also present an issue for ingested glucose, albeit maybe not as important as gut transport. Sites involved in the regulation of muscle glucose uptake during this process include glucose delivery to muscle from the capillary bed and across the interstitium, transport of glucose into the muscle by GLUT4, and phosphorylation of glucose within the muscle by hexokinase II (HKII). Muscle blood flow, capillary recruitment, and transport across the endothelial wall determine glucose movement from the blood to the interstitium, whilst plasma membrane GLUT4 content determines glucose transport into the cell, and muscle HKII activity determines the capacity to phosphorylate glucose and enable cell processing (Kusters and Barrett, 2016; Messina et al. 2015). Figure 3 illustrates the processes of glucose delivery and transport.

Since glucose uptake is the product of blood flow and the arteriovenous glucose difference, the increase in blood flow is quantitatively the major contributor to the exercise-induced increase in muscle glucose uptake. This is particularly apparent since an increase in blood glucose concentration has been demonstrated to equate to a parallel increase in interstitial glucose concentration during exercise, and that skeletal muscle contraction results in an increase in the diffusion coefficient of glucose within the interstitial space (MacLean et al. 1999). The authors also found higher lactate concentrations within the interstitial space compared to that within the venous and arterial plasma. This suggests that the diffusion across the interstitial space, particularly during exercise, may be a possible barrier for glucose transport. In addition to the large increase in flow to contracting skeletal muscle during exercise, there is also recruitment of capillaries which increases the available surface area for glucose delivery towards the interstitium via GLUT1.

The regulation of blood flow to skeletal muscle is tightly coupled to the metabolic demand for oxygen with a change in oxygen requirement leading to a proportional change in blood flow. The precisely regulated control of blood flow serves to minimize the work of the heart while ensuring adequate oxygen supply to the working muscle. Skeletal muscle blood flow can increase up to 20-fold from rest to intense, dynamic exercise (Andersen and Saltin 1985). Given the limitation in maximal cardiac output, the heart can only supply a fraction of working muscles with maximal blood flow, and during hard aerobic exercise involving larger muscle mass, vascular conductance has to be well regulated or blood pressure may fall (Gleimann et al. 2019). The overall regulation of skeletal muscle blood flow is achieved through a balance between, on one hand, sympathetic vasoconstriction and circulating vasoconstrictors, and on the other hand vasodilators derived from cells in the skeletal muscle tissue.

It is established that elevations in insulin lead to an increase in blood flow and thus glucose uptake (Baron 1994; DeFronzo et al. 1981). This process involves endothelium-derived nitric oxide (NO) which is known to regulate the transport of insulin and uptake of glucose by several tissues including skeletal muscle. Nitric oxide enhances flow-mediated vasodilation and improves the delivery of nutrients such as glucose. Conversely, both high glucose and insulin stimulate the transport of L-arginine and increase NO production in vascular endothelial cells (Sobrevia et al. 1996). In addition, insulin has been shown to stimulate skeletal muscle blood flow and enhance vasodilation by increasing NO release (Roy et al. 1998). Taken together, studies have demonstrated that elevations in insulin concentrations lead to an increase in blood flow which is mediated through NO production, leading to increased glucose uptake. What has been known for many years is that muscle glucose utilisation increases despite decreased insulin, because exercise causes translocation of GLUT4 glucose transporters from a different pool than insulin, and the exercise-induced signaling of glucose utilisation is independent of insulin signaling. Furthermore, increased peripheral blood flow augments total insulin delivery to muscle and so compensates at least in part for the decreased plasma insulin concentrations. This also explains why the insulin and exercise effects are additive (Marliss and Vranic 2002).

Blood glucose concentration is another important determinant of muscle glucose uptake during exercise. Since glucose uptake across an exercising limb follows saturation kinetics with a Km found to be 10 mM during knee-extension exercise in humans (Richter and Hargreaves 2013), changes in plasma glucose concentration translate almost directly into proportional changes in leg glucose uptake. During prolonged exercise, liver glucose output is reduced and hypoglycemia can limit muscle glucose uptake (Felig et al. 1982). In contrast, increasing arterial glucose availability, by ingestion of CHO-containing beverages or by infusion, results in increased muscle glucose uptake and oxidation during prolonged exercise (Jeukendrup et al. 1999; MacLaren et al. 1999). In terms of high-intensity exercise, Schrader et al. (2016) found no significant influence of CHO supplementation on RER despite significantly increase insulin and glucose concentrations. In addition, RER remained significantly higher during the recovery period for the CHO supplementation group. This suggests that elevated insulin and potential increases in blood flow due to higher blood glucose may be important.

It has been known for many years that muscle glucose transport is carrier mediated and that the specific transport proteins responsible for insulin- and contraction-stimulated glucose transport in skeletal muscle have been identified (Charron et al. 1989). GLUT1 is generally expressed and is thought to be responsible for the basal uptake of glucose. Hence it is depicted in Fig. 3 on the endothelial surface for the transport of glucose into the interstitium as well as being responsible for basal levels of glucose uptake in the muscle (Olson and Pessin 1996). GLUT4 on the other hand is expressed most abundantly in adipose tissue and skeletal muscle.

**Fig. 4:**
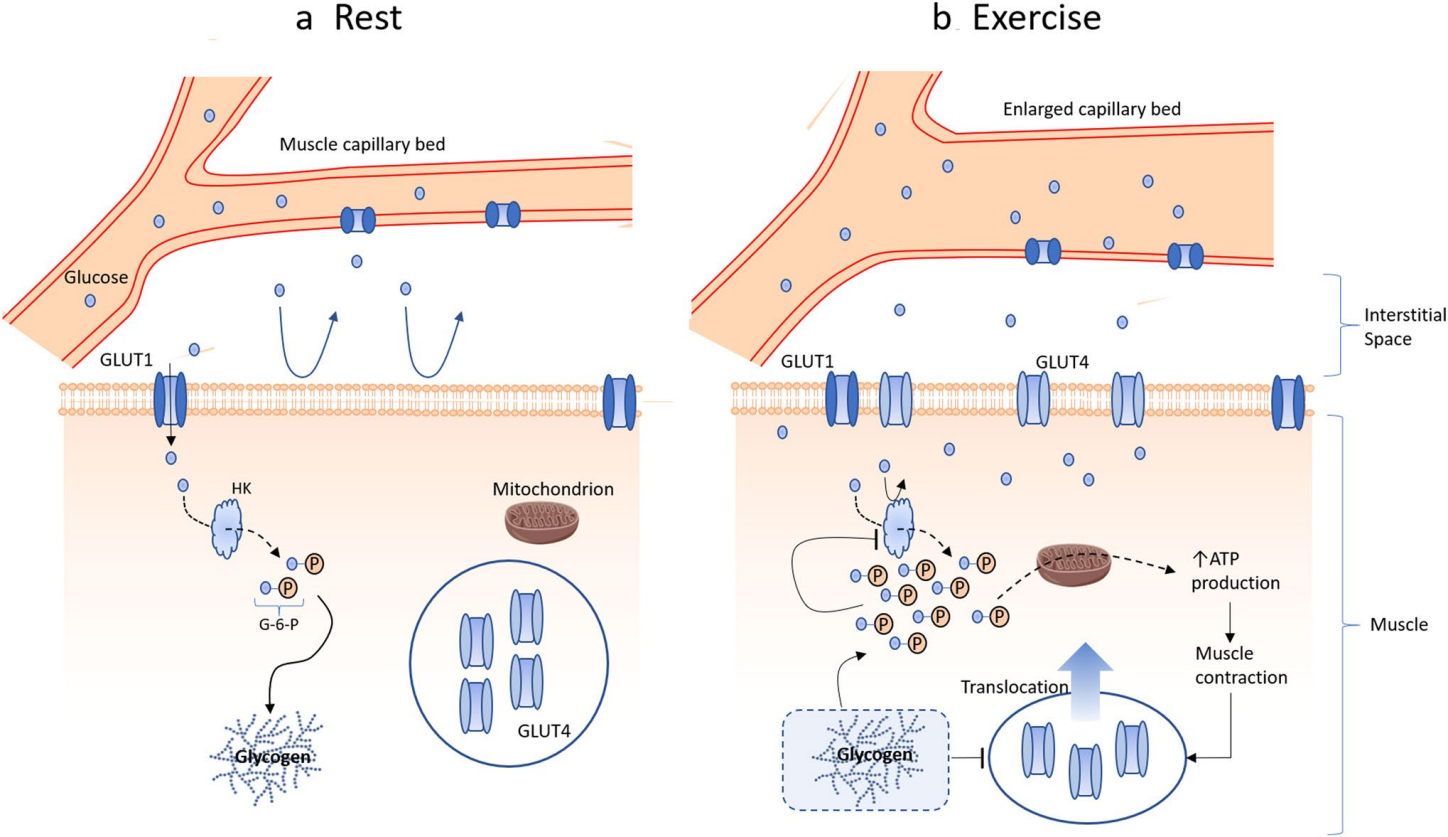
Schematic illustrating the GLUT4 translocation process during both **a** rest and **b** exercise

GLUT4 resides in storage vesicles within the cell and are directed to fuse with the plasma membrane through both an increase in circulating blood glucose and exercise per se (Richter and Hargreaves 2013). In the case of an increase in blood glucose, insulin concentrations are elevated. Insulin binds with its receptor and results in an increase in protein kinase B (Akt), thereby promoting GLUT4 translocation. Additionally, exercise per se also stimulates GLUT4 translocation via increases in AMP-activated protein kinase (AMPK) due to changes in the ATP: ADP ratio (Richter and Hargreaves 2013), as well as via enhanced intracellular calcium (Ca^+^) (McCormack and Denton 1990), reactive oxygen species (ROS) production (Powers et al. 2020) and Nitric oxide (NO) production (Tsukiyama et al. 2016). The GLUT4 translocation process is complex, involving numerous cellular processes (Fig. 4). In skeletal muscle, the movement of transporters occurs by the exocytosis, trafficking, docking, and fusion of GLUT4-containing storage compartment or “vesicles” into the cell-surface membranes. When skeletal muscles are stimulated simultaneously by contraction and insulin treatments, there are additive effects on glucose transport. Consistent with these findings, the combination of exercise and insulin can have additive effects on GLUT4 translocation to the sarcolemma. These data support the concept that there are different mechanisms leading to the stimulation of muscle glucose transport by exercise and insulin. However, for a more detailed exploration on this topic it is worth consulting Egan and Zierath (2013), Mul et al. (2015), and Richter and Hargreaves (2013).

The relationship between GLUT4 and muscle fibre type has been reported and signified that Type1 fibres have a slightly greater content of GLUT4 than Type IIA and Type IIX in the vastus lateralis though not in soleus or triceps brachii (Daugaard and Richter 2004). These findings show that GLUT4 expression is not dependent on fibre type per se but is muscle dependent, otherwise similar results would accrue between vastus and triceps brachii. However, what has been definitively shown is that athletes have greater levels of GLUT4 than untrained individuals (Andersen et al. 1993), and also that GLUT4 content can be increased appropriate training (Daugaard et al. 2000; Houmard et al. 1995). Daugaard et al (2000) found that after 2 weeks of one-legged low-intensity training a 26% increase in total skeletal muscle membrane GLUT4 content in Type I fibres but not in Type IIA or Type IIX. These data reflect the low level of activity during training in which predominantly Type I fibres are recruited. It is possible that a training intensity in which all fibres were recruited could show similar enhancement of GLUT4 across all fibre types.

Once inside the muscle cell, glucose is immediately converted to glucose-6-phosphate (G-6-P) using HKII. This process is irreversible and so once glucose enters a cell it cannot ‘escape’. The rapidity of conversion of glucose to G-6-P is a potential barrier to the rate of uptake of glucose. HKII is the second step of glucose utilization in insulin-sensitive tissues and may be considered rate-limiting under conditions where glucose transport is maximally stimulated (Katz et al. 1991; Ren et al. 1993). Skeletal muscle HKII mRNA levels and enzymatic activity are decreased when insulin is low or when insulin signaling is impaired. In contrast, HK II mRNA levels and enzyme activity are increased by exercise (O’Doherty et al. 1994).

Inhibition of HKII is achieved through product inhibition by G-6-P. If G-6-P cannot be removed at a sufficient rate during exercise as in the case of maximal intensity bouts when glycogenolysis is favoured, a build-up of G-6-P results. The effect is product inhibition of HKII with a resultant attenuation of glucose uptake. At the start of exercise, there is evidence of an elevation of glucose within a muscle which demonstrates inhibition of HKII (Richter and Hargreaves 2013). This is probably due to preferential use of muscle glycogen at the start of exercise thereby elevating G-6-P; although as the exercise duration increases enhanced glucose uptake is observed. Consequently, a concomitant reduction in intra-muscle glucose, probably due to reduced G-6-P and hence increased HKII activity, results. It is also worth noting that starting exercise with a high muscle glycogen content produces a reduction in glucose uptake since muscle glycogenolysis is favoured, and as the glycogen levels are reduced with exercise there is an increase in glucose uptake. It would appear that HKII is a regulatory factor for glucose uptake either during maximal exercise or at the onset of exercise but unlikely at other time points in steady-state exercise.

So, which of the processes of delivery, transport, and metabolism are limiting factors for glucose uptake and oxidation in a muscle cell? The answer appears to be dependent on the level of exercise intensity. At maximal exercise intensity, delivery is clearly an issue due to cardiac output being unable to fully meet the demands, but equally inhibition of HKII by G-6-P would result in a reduced ability of muscle to take up and utilize the exogenous glucose. During steady-state bouts of exercise, where blood flow matches the oxygen demands of the cell, it is likely that delivery of glucose may be enhanced by increasing insulin through glucose intake and thereby promoting increased capillary flow.

## Hormonal response to CHO ingestion and infusion during exercise

The complexity and diversity of the entire hormonal response to exercise are too large to be covered in detail here, and so the focus on the metabolic effects of exercise in relation with the endocrine response will be confined to insulin, glucagon, and the sympathoadrenal system. An early but classic description of the hormonal response to exercise was comprehensively reviewed by Galbo (1983), and the findings are still relevant today.

The sympathoadrenal system releases the hormones epinephrine, norepinephrine and cortisol, and although norepinephrine is often referred to as a hormone it is more akin to the action of a neurotransmitter. Increases in sympathetic nervous activity as a function of exercise have been reported to be linked to increased activity of the motor cortex of the brain (Jansson and Kaijser 1987; Victor et al. 1995). The effects of exercise on circulating catecholamine release can be summarized as follows: exercise induces an increase in catecholamines that is observed across a wide range of exercise modalities (Galbo et al. 1975), is exercise-intensity dependent (Galbo et al. 1975; Jansson and Kaijser 1987) and is diminished with training (Winder et al. 1978; Phillips et al. 1996), being lower in trained compared with untrained individuals (Kjaer and Galbo 1988). The exercise-induced increase in catecholamine concentration is sufficient to stimulate glycogenolysis in both the liver (Kjaer et al. 1993) and skeletal muscle (Richter et al. 1981; Spriet et al. 1988).

During exercise, the effects of increasing plasma epinephrine concentration on the metabolic response in muscle report an increase in CHO utilisation (Richter et al. 1981; Chasiotis and Hultman 1985; Febbraio et al. 1998). The effects of adrenergic stimulation on CHO metabolism have also been examined in relationship with glucose uptake by skeletal muscle. Infusion of epinephrine has been reported to decrease glucose uptake (Watt et al. 2001), although the rate of CHO utilisation is enhanced, thereby demonstrating a shift towards intracellular CHO utilisation and away from extracellular glucose. Studies in which a CHO is ingested or glucose infused have demonstrated that catecholamines are somewhat suppressed with increases in blood glucose and insulin (MacLaren et al. 1994, 1999). The greater the blood glucose, the higher the insulin and the lower the catecholamines.

Moderate exercise (60–75% ${\dot{\text{V}}}$O_2max_) is an example of euglycemic homeostasis in which there is a precise match between the increases in glucose utilisation and glucose production. This is considered to be feedback-regulated by signals associated with the increased demand by the exercising muscles, causing responses that increase glucose production to match glucose utilisation. During exercise, insulin secretion is inhibited below fasting levels by adrenergic receptor activation, both via the sympathetic innervation of the islets and by circulating catecholamines (Marliss and Vranic 2002). The decrease of insulin secretion is important because it increases glucose production by the liver by sensitizing it to glucagon (Zinker et al. 1994). It is established that the decreases in insulin and unchanged or increased glucagon account for the corresponding increases in glucose production (Wasserman et al. 1989). Thus, the ratio of glucagon to insulin is the major regulator of glucose production during moderate exercise. Catecholamines play a role in increasing glucose production, through gluconeogenesis, only during prolonged exercise > 2 h duration (MacLaren et al. 1999). Their increase during 40 min of moderate exercise are modest and predicted to have limited effects. When exercise is accompanied by ingestion or infusion of CHO/glucose, the normal attenuation of insulin and elevation of glucagon are reversed (MacLaren et al. 1994, 1999). In fact, with an infusion of glucose to attain hyperglycaemia, the insulin levels may be elevated fourfold with ingestion (MacLaren et al. 1994) to six or seven-fold with infusion (MacLaren et al. 1999). Indeed, in our recent publication with insulin infusion a 20-fold increase in insulin concentration was achieved (Mohebbi et al. 2020). Schrader et al. (2016) also observed a fourfold increase in insulin concentration despite an almost unchanged RER during high-intensity exercise. The consequence is that CHO oxidation and use is promoted whilst fat oxidation is attenuated, and as mentioned in the previous section could be in some part due to enhanced capillarisation.

## Oxidation of ingested sources of CHO: dose and type of CHO

The progression from CHO ingestion to its oxidation and use as an energy source is a complex process, one which Rosset et al. (2017) suggest is potentially a limiting factor for CHO use during exercise. One of the earliest investigations into the assessment of exogenous CHO oxidation employed the radioactive tracer ^14^C and observed that when 32 g of CHO was ingested during exercise at 66%${\dot{\text{V}}\text{O}}_{{{\text{2max}}}}$, the exogenous CHO oxidation amounted to 5% of total CHO oxidized and the rate was 0.03 g/min (Costill et al. 1973). Another study, also using ^14^C, found a slightly higher finding of 0.7 g/min when participants exercised at 50%${\dot{\text{V}}\text{O}}_{{{\text{2max}}}}$ for 60 min (Van Handel et al. 1980). These rather low values of the rate of exogenous oxidation contrast sharply with other reported findings using ^13^C isotopes is subsequent years and may have been an anomaly in the techniques applied at that time as well as the lower exercise intensities employed. The results from some of the earlier studies using ^13^C and exercise provided rates of exogenous CHO oxidation between 0.5 and 1.1 g/min; values tenfold higher than those observed with ^14^C (Decombaz et al. 1985; Guezennec et al. 1989; Hawley et al. 1992; Massicotte et al. 1986, 1989, 1990; Pallikarikas et al. 1986; Pirnay et al. 1977).

Many subsequent and more recent investigations have produced similar findings to the classical earlier studies i.e. that the rates of exogenous CHO oxidation seem to be around 0.7–1.2 g/min. However, some types of CHO are more readily oxidized than others (Currell and Jeukendrup 2008). Clearly, glucose is the yardstick by which other CHO sources may be compared since it requires no digestion and is readily absorbed. Consequently, many investigations have used comparison with glucose as a measure of their efficacy. There are clear findings that maltose, sucrose, maltodextrins, and glucose polymers result in very similar rates of exogenous oxidation (Jeukendrup and Jentjens 2000). Even high molecular weight glucose polymers (Rowlands et al. 2005) and soluble starch (Hawley et al. 1991) are oxidized to the same extent as glucose. So, it appears that digestion and absorption of these various CHO sources is not an issue, although any highly concentrated form of a CHO may affect gastric emptying and cause GI disturbances.

Fructose, on the other hand, has readily exhibited an inferior rate of oxidation in most reported studies (Jandrain et al. 1993; Massicotte et al. 1986, 1989). The reduced rate in these studies is consistently reported to be approximately 25%. This is in part due to the slower absorption of fructose across the intestinal cells due to the diffusion dependent on a concentration gradient, as well as the fact that almost all the fructose is taken up by the liver in a first-pass (Tappy and Le 2010). The liver then converts the fructose to glucose and lactate for further distribution.

Likewise, galactose has been reported to have a significantly lower rate of oxidation when compared with glucose (Leijssen et al. 1995). The authors determined a 50% reduction in exogenous oxidation (0.41 g/min) compared with glucose (0.85 g/min) when cycling at 65% ${\dot{\text{V}}\text{O}}_{{{\text{2max}}}}$ for 2-h. More recently O’Hara et al. (2012) observed higher rates in exogenous oxidation for glucose in the first 90 min of exercise at 60% ${\dot{\text{V}}\text{O}}_{{{\text{2max}}}}$ (peak oxidation 0.68 g/min) but higher rates between 90 and 150 min for galactose (peak oxidation 0.44 g/min). However, a single 75 g bolus of CHO was provided rather than a continuous supply. It is likely that the faster absorption of glucose resulted in the elevated oxidation earlier on whereas the slower uptake of galactose resulted in higher oxidation after 90 min.

It is likely that oxidation of a single exogenous CHO is limited to approximately 60 g/h because there is a limitation in the rate of intestinal absorption of that CHO type (Jeukendrup 2004). It is suggested that by feeding a single CHO source (e.g. glucose, fructose or maltodextrins) at high rates, the specific transporter proteins that aid in absorbing that CHO from the intestine become saturated. Once these transporters are saturated, feeding more of that CHO will not result in greater intestinal absorption and increased oxidation rates. Shi and colleagues (1995) suggested that the ingestion of CHO that use different transporters might increase total CHO absorption. Subsequently, a series of studies using different combinations of CHO (in effect glucose and fructose at a ratio of 2:1) to determine their effects on exogenous CHO oxidation were undertaken (Jentjens and Jeukendrup 2005). In one study, participants ingested a drink containing glucose and fructose, whereby the glucose was ingested at a rate of 72 g/h and fructose at a rate of 36 g/h (i.e. 2:1 ratio), whilst the controls were ingestion of glucose at a rate of 72 g/h and 108 g/h. Ingestion of glucose at 72 g/h and 108 g/h resulted in oxidation rates of approximately 0.80 g/min, which are as previously reported. However, ingestion of glucose plus fructose resulted in a rate of exogenous CHO oxidation amounting to 1.27 g/min, an increase of 45% compared with the glucose (Jentjens et al. 2004). In a proceeding investigation, the same authors observed the highest rates with a mixture of glucose and fructose ingested at a rate of 144 g/h. With this regimen, exogenous CHO oxidation peaked at 1.75 g/min, which is 75% greater than what was previously thought to be the maximum (Jentjens and Jeukendrup 2005). Taken together, these data would suggest that intestinal absorption is a ‘barrier’ to oxidation of exogenous CHO.

More recently, investigations have been conducted on CHO sources which have been encapsulated in a hydrogel. The rationale is that encapsulation of high CHO dose would not compromise gastric emptying and thereby result in reduced GI discomfort. Although in its infancy, early findings do not appear to show higher rates of exogenous CHO oxidation compared with glucose or maltodextrin (Baur et al. 2019; McCubbin et al. 2020). It should be mentioned that these studies did not examine exogenous oxidation but rather total CHO oxidation. There is scope to explore the impact of such encapsulation of a CHO source with labelled ^13^C and thereby determine the rates of exogenous oxidation, although to date no such investigation has been reported.

Any factors which purport to enhance intestinal absorption during exercise would seem eminently sensible to pursue. To this end, a recent study examined the potential efficacy of 4 weeks of probiotic supplementation on subsequent oxidation of exogenous maltodextrin (Pugh et al. 2020). The results highlighted a small but significant increase in exogenous CHO oxidation after supplementation compared with placebo, although the authors were unable to confirm whether intestinal absorption was a factor. Furthermore, the rates of oxidation after supplementation or placebo were 0.8 and 0.7 g/min, respectively; values not dissimilar from the plethora of other data.

## Oxidation and utilisation of infused glucose

The hyperglycaemic glucose clamp technique was devised by DeFronzo et al. (1979) to quantify β-cell sensitivity to glucose. In essence, the procedure requires plasma glucose to be elevated to a selected level (typically above 10 mM) by infusion, followed by an infusion rate of glucose designed to ‘clamp’ the plasma levels to that value (see MacLaren et al. 1999). Thereby, the rate of glucose infused is an index of glucose metabolism in which the glucose is either oxidized and/or stored (as glycogen or fat). Although the focus of this technique has been mainly applied to clinical settings in relation to diabetes and metabolic syndrome (Vandemeulebroucke et al. 2010), as well as sepsis (White et al. 1987), colorectal cancer (Copeland et al. 1988), and multiple organ failure (Green et al. 1990), a few investigations have also been reported in an exercise context (Coyle et al. 1991; Hawley et al. 1994; MacLaren et al. 1999; Malone et al. 2019, 2020; Mohebbi et al. 2020; Weltan et al. 1998).

The focus of the studies employing the hyperglycaemic clamp during exercise has been to report total CHO oxidation as well as utilization of the infused glucose. Additionally, the effects on circulating hormones and muscle glycogen use have been reported in some of the investigations. An important factor to consider with hyperglycaemic clamp studies is that by clamping at glucose concentrations of 10–12 mM there is complete cessation of liver glucose output i.e. the blood glucose levels are maintained entirely due to exogenous infused CHO and do not arise from the liver output via glycogenolysis or gluconeogenesis (Hawley et al. 1994).

Typically, the rate of GUR with infusion lies between 1.9 and 2.6 g/min and reflects the disposal of the infused glucose (Coyle et al. 1991; Hawley et al. 1994; MacLaren et al. 1999; Malone et al. 2019, 2020; Weltan et al. 1998). Most, but not all, of the glucose is likely to be oxidized in muscle. These values are significantly higher than those of exogenous oxidation observed in studies involving ingestion, and appear to be the highest rates observed in exercising humans. It is unlikely that total carbohydrate oxidation rates greater than ~ 3 g/min will be seen. However, what is unknown is the proportion of the GUR which is actually oxidized and so a direct comparison is not possible. Such an investigation was achieved by Hawley et al. (1994) who calculated that 60% of the infused glucose was actually oxidized during the exercise (the other 40% being disposed probably as muscle glycogen synthesis). If this is correct, the amount of infused glucose actually oxidized would be approximately 1.8 g/min.

Recently, we employed both hyperglycaemia and hyperinsulinaemia during exercise and determined that the GUR increased to 3.1 g/min compared with 2.4 g/min with hyperglycaemia alone (Mohebbi et al. 2020). This represents an approximate 25% higher GUR. The only difference between the trials was that insulin levels were 2.5 times higher with insulin + glucose infusion compared with glucose alone. Exercise is associated with marked increases in blood flow and capillary surface area to working muscle, which in turn leads to increased uptake of glucose by exercising muscle. DeFronzo et al. (1981) demonstrated that when 30-min of mild exercise (40% ${\dot{\text{V}}\text{O}}_{{{\text{2max}}}}$) was combined with hyperinsulinaemia (~ 75 U/ml), leg blood flow increased approximately nine-fold and glucose uptake increased markedly for the same rate of insulin infusion. The interpretation of their findings was that the increase in glucose uptake (for the same insulin level) was mediated by increased blood flow to, and increased capillary surface area in, the exercising muscles. This interpretation was supported by close correlations between the changes in blood flow and glucose uptake, a fact also observed by Baron (1994). Exercise and insulin are thus shown to interact synergistically in the control of glucose uptake (DeFronzo et al. 1981; Wasserman et al. 1991). So, it appears that the ~ 25% ‘extra’ CHO utilized under hyperglycaemia with hyperinsulinaemia is probably due to enhanced blood flow and glucose transport to the working muscle.

## Muscle glucose oxidation during exercise

Muscle glycogen is the predominant CHO source during moderate to intense exercise, and the rate of degradation is related to the relative exercise intensity. Increased glycogenolysis during exercise occurs via activation of glycogen phosphorylase, which reflects alterations in [Ca^i^], [Pi], cAMP-dependent β-adrenergic stimulation, and allosteric modulation by AMP and inosine monophosphate (IMP) (Hargreaves 2006). Higher rates of glycogenolysis occur when initial muscle glycogen concentrations are high, but as exercise proceeds, degradation rates parallel declining glycogen levels and associated glycogen phosphorylase activity. The use of muscle glycogen during exercise with CHO intake is equivocal. Some studies report muscle glycogen sparing (Stellingwerff et al. 2007; Tsintzas et al. 1995) whilst others observe no sparing (Coyle et al. 1986; Hawley et al. 1994; MacLaren et al. 1999).

Skeletal muscle CHO oxidation is promoted when CHO sources are ingested or infused (MacLaren et al. 1994, 1999). This is a result of increases in insulin and reductions in glucagon and catecholamines favouring glucose disposal and attenuating fat oxidation. This coordinated hormonal response, in our findings, does not result in sparing muscle glycogen but rather promotes all aspects of CHO use.

The fact that trained individuals have a lower rate of glucose oxidation than untrained individuals would suggest that, in part, delivery to the muscle by circulation could be a compromising factor. However, other factors such as glucose transport across the muscle membrane and oxidation of the glucose are important considerations too. It is known that endurance training reduces muscle glucose uptake during exercise (Coggan et al. 1990; Jansson and Kaijser 1987), an adaptation that is associated with reduced sarcolemmal glucose transport and GLUT4 translocation (Richter et al. 1998), at least during exercise at the same absolute exercise intensity. When exercise is performed at the same relative intensity, differences between untrained and trained are smaller or nonexistent (Bassami et al. 2007). In a study where ^13^C glucose was ingested in trained and untrained participants during exercise at 60% ${\dot{\text{V}}\text{O}}_{{{\text{2max}}}}$, Jeukendrup et al. (1997) observed similar rates of exogenous oxidation between the groups; the only difference between the groups was elevated fat oxidation in the trained participants. During dynamic knee extension exercise at peak power output, glucose uptake has been observed to be higher in the trained limb as is GLUT4 expression and oxidative capacity (Kristiansen et al. 2000). So, it appears that the skeletal muscle GLUT4 level does correlate with the capacity for glucose uptake during very intense exercise but not moderate exercise.

In a recent publication, we reported that a 3-days high fat or high CHO diet presented with a significant increase in fat or CHO oxidation, respectively, during exercise with glucose infusion (Malone et al. 2020). Clearly, there were no effects of a training programme to warrant these changes in such a short time. Examination of the insulin data demonstrated that 3 days of CHO loading resulted in a significantly higher concentration than with 3 days of low CHO intake, and, furthermore, that the resultant was greater CHO oxidation at the same exercise intensity. It would appear that enhanced fatty acid uptake and oxidation by skeletal muscle is up-regulated by short periods of dietary manipulation and that this is maintained despite hyperglycaemia. In other words, ‘fat rules’ i.e. any elevation of fat oxidizing capability by diet and/or training is likely to ‘dominate’ muscle CHO oxidation. We feel that further work in this area is warranted to elucidate the likely mechanisms.

Another factor that needs to be considered is that of trade-off between endurance training and ingestion of a high CHO diet for performance enhancement. It appears that in such a case when a high CHO diet is ingested over a 28-days period, the rate of exogenous CHO oxidation is significantly enhanced by approximately 13% (Cox et al. 2010). It would appear that whilst endurance training leads to a reduction in CHO oxidation, a high CHO diet can reverse these effects by increasing CHO oxidation rates. This paradoxical scenario must be considered by both untrained and trained individuals when undertaking exercise programmes.

## Conclusion

Ingested CHO sources are not oxidised by skeletal muscle in the same proportion as they are ingested. By implication, this means that there are some ‘barriers’ in the delivery of the glucose to muscle and its subsequent oxidation within the mitochondria. The first potential ‘barrier’ is that of gastric emptying from the stomach which is compromised to an extent if solutions of high osmolarity are ingested, although training the gut may alleviate this somewhat. Beyond the stomach is the issue of digestion and subsequent absorption of the CHO source. Digestion does not appear to be an issue even with glucose polymers and polysaccharides, whereas absorption across the intestine does present a significant ‘barrier’ in so far as there is a limit to the rate of absorption dependent on the glucose transporters. This is particularly noteworthy with the limited transporters for fructose uptake but is also a problem vis a vis glucose absorption. It is within this context that forthcoming research could explore upregulation of SGLT1 with diet; the only study apparently in this instance is on mice rather than humans (Ferraris et al. 1992). Having been absorbed, the glucose and fructose need to bypass the liver which presents no issues with glucose, although most of the fructose is taken up by the liver. The liver can then convert some of the fructose to glucose, which is then transported to muscle. Another factor that should be raised is that splanchnic blood flow is diminished during exercise and is intensity dependent.

Glucose, once away from the gut, is transported to the exercising skeletal muscle. Skeletal muscle blood flow is significantly increased during exercise and this is more so with trained individuals. Furthermore, it is apparent that an elevated insulin concentration may impact on blood flow. Studies in which hyperinsulinaemia are evident clearly show both an increased blood flow and enhanced muscle glucose uptake. To enter a muscle cell, glucose is required to be transported across the sarcolemma and T-tubules. GLUT4 translocation from vesicles within the cell to fuse with the membrane is stimulated by exercise and by elevated insulin. Any means of increasing GLUT4 is more than likely to promote glucose uptake and oxidation. Training apparently achieves this in regard to intense exercise but not moderate exercise. A longitudinal training study on the effect of training (and possibly diet) on GLUT4 expression at the same absolute and relative exercise intensities would be pertinent.

Endurance training studies invariably report enhanced fat oxidation and diminished CHO oxidation with muscle glycogen sparing at the same absolute exercise intensity. The consequence of these training studies is a diminished oxidation of glucose either from liver or from exogenous sources (Phillips et al. 1996). It appears that endurance training attenuates glucose oxidation in favour of fat oxidation without recourse to modifications in gut absorption. In this regard, it is likely that hormonal and intracellular modifications play important roles.

Future investigations using glucose infusion with and without insulin infusion could perhaps focus on muscle blood flow, glucose transport into the interstitium, GLUT4 translocation and modifications in HKII activity. The use of ingested (or infused) labelled glucose would help determine the degree of exogenous use. Finally, it is possible to explore the variations of exercise intensity with infused glucose on the regulation of CHO oxidation. We have explored so-called gross changes in our studies without recourse to changes in blood flow or muscle metabolites.

In summary, whereas ingestion maybe limited by factors such as gastric emptying (if the CHO source is hypertonic), absorption across the intestine (notably fructose but also glucose), by passing the liver (notably fructose), and by transport across the sarcolemma, infusion studies reveal possible limitations with blood flow and glucose uptake at the muscle. Table 1 highlights some of the potential ‘barriers’ to glucose oxidation from exogenous sources of glucose by muscle during exercise. Paradoxically, endurance training, although it enhances muscle blood flow actually attenuates glucose uptake and oxidation due to enhanced fat oxidation. It is probable that the hormonal changes which occur during exercise promote greater fat oxidation, which in turn attenuates CHO oxidation and that these changes are enhanced by endurance training.

**Table 1:** Some potential ‘barriers’ to carbohydrate ingestion and glucose infusion

Potential ‘Barrier’	Carbohydrate ingestion		Glucose infusion	
	Issues	Potential solutions	Issues	Potential solutions
Gastric emptying	GI problems with high osmolality/hypertonic solutions	Isotonic or ↓CHO content solutions (> 60 min) Trainable	Not applicable	
Intestinal absorption	Probable major barrier ↓Transportation (saturation of SGLT1 and GLUT5)	Upregulation with ↑CHO diet Multiple CHO transporter	Not applicable	
Bypass liver	Uptake of Fructose by the liver Fructose → Glucose delay	Glucose or Multiple CHO transporters	Not applicable	
Splanchnic blood flow	↓during exercise ↑gut permeability	↑flow in trained individuals	Not applicable	
Muscle blood flow	Limited Q̇ Unable to provide all muscles with maximal flow during whole body exercise	↑with training adaptations	Limited Q̇ Unable to provide all muscles with maximal flow during whole body exercise	↑with training adaptations Possible ↑with high insulin
Muscle uptake	↓ Blood flow and GLUT4	↑with training adaptations ↑with increased insulin		↑with training adaptations ↑with insulin stimulation greater than ingested CHO
Muscle oxidation	CHO oxidation ↓with endurance training Limited number of protein carriers	Promoted by CHO intake Potential to supplement with probiotics to ↑oxidation	↓with endurance training	Significantly ↑with infusion

## Abbreviations

Akt: Protein kinase B
AMPK: AMP-activated protein kinase
Ca^+^: Calcium
CHO: CHO
GLUT: Glucose transporter
GUR: Glucose utilisation rate
IMP: Inosine monophosphate
NO: Nitric oxide
ROS: Reactive oxygen species
SGLT1: Sodium-dependent glucose transporter
${\dot{\text{V}}}$O_2max_: Maximal oxygen uptake
${\dot{\text{V}}}$O_2peak_: Peak oxygen consumption
